# Supplementary material for: Content-rich biological network constructed by mining PubMed abstracts
Source: BMC Bioinformatics. 2004 Oct 8;5:147. doi: 10.1186/1471-2105-5-147 (PMC528731; doi:10.1186/1471-2105-5-147)
Supplement: Additional File 5 — The original Chilibot query results of the term "long-term potentiation (LTP)" and 22 other terms, limiting the latest references analyzed to the years 1990, 1995, 2000, and 2004. [file 1471-2105-5-147-S5.bz2 › chilibotAdditionalFile5/ltp2000/html/ARC_CAMKII.html]

 


 **ARC** and **CAMKII** 
  
Found 1 abstracts in PubMed,  **1 abstracts were retrieved and analyzed**.  


---

 Search Google  |
 PDF files only 
|  EDU domain only 

---

**Interactive relationship** (e.g. stimulation, inhibition, etc)

**Parallel relationship** (e.g. studied together, co-existance, homology, etc.)

- We show that a fraction of alpha  **CaMKII** , InsP3R1, and  **Arc**  mRNAs present in synaptosomes is indeed associated with polysomes.  Ref: 10783400 J Neurosci, 2000
- Moreover, we show that polysomal association of alpha  **CaMKII**  mRNA... increases with depolarization of the synaptosomal membrane.  Ref: 10783400 J Neurosci, 2000
- To address this point we analyzed the polysomal association of the mRNAs for the alpha subunit of calcium calmodulin dependent protein kinase II alpha  **CaMKII** , for type 1 inositol, 5 trisphosphate receptor InsP3R1 and for the activity regulated cytoskeleton associated protein  **Arc**  in a synaptosomal preparation devoid of contaminating material from neuronal and glial perikarya.  Ref: 10783400 J Neurosci, 2000
